# Preliminary effects of mobile computerized cognitive training in adults with mild cognitive impairment: interim analysis of a randomized controlled trial

**DOI:** 10.1186/s40359-025-02458-w

**Published:** 2025-03-05

**Authors:** Drin Ferizaj, Oskar Stamm, Luis Perotti, Eva Maria Martin, Kathrin Finke, Carsten Finke, Tilo Strobach, Anika Heimann-Steinert

**Affiliations:** 1 Department of Geriatrics and Medical Gerontology (Forschungsgruppe Geriatrie), Charité – Universitätsmedizin Berlin, corporate member of Freie Universität Berlin and Humboldt-Universität zu Berlin, Reinickendorfer Straße 61, Haus 7, 2. OG, Berlin, 13347 Germany; 2 Memory Center, Department of Neurology, Jena University Hospital, Jena, Germany; 3 Department of Neurology, Charité – Universitätsmedizin Berlin, corporate member of Freie Universität Berlin and Humboldt-Universität zu Berlin, Sauerbruchweg 5, Berlin, 10117 Germany; 4 Department of Psychology, ICAN Institute for Cognitive and Affective Neuroscience, Medical School Hamburg, Am Kaiserkai 1, Hamburg, 20457 Germany

**Keywords:** Mild cognitive impairment, Cognitive training, Computerized training, Mobile intervention, Randomized controlled trial

## Abstract

Computerized cognitive training (cCT) is a non-invasive treatment strategy in which individuals repeatedly practice computer-based tasks targeting specific cognitive functions. While traditional face-to-face cognitive training is costly and accompanied by long waiting times, mobile cCT can improve the healthcare situation due to its accessibility and versatility. This interim analysis of the NeuroNation MED Effectiveness Study (NeNaE) explores the initial effects of a mobile, gamified cCT (NeuroNation MED) on cognitive and psychosocial outcomes in adults diagnosed with mild cognitive impairment (MCI; ICD-10 code F06.7). In the intention-to-treat analysis, the intervention group (IG) performed 12 weeks of training with the NeuroNation MED app, while the control group (CG) served as a waiting group. This interim analysis included the first 50 participants (IG: *n* = 36; CG: *n* = 14). The primary outcome was the index score of the Neuropsychological Assessment Battery Screening Module (S-NAB). Alpha spending resulted in an alpha level of 0.00305 for this interim analysis. This analysis provided preliminary evidence suggesting potential benefits of the intervention, with the IG demonstrating a significant improvement in the S-NAB index score (*t*(35) = 3.17, *p* = 0.0028, Cohen's *d* = 0.53). However, ANCOVA revealed no significant group differences between IG and CG in the S-NAB index score (*F*(1, 47) = 5.39, *p* = 0.025, *η*_*p*_^*2*^ = 0.125). Additionally, the IG showed a significant mean decrease in subjective cognitive failures measured by the CFQ-D (*MD* = -10.56, *SD* = 18.82, *t*(35) = 3.21, *p* = 0.0029, Cohen's *d* = 0.53). ANCOVA showed no significant group difference in CFQ-D post-test scores when controlling for pre-test scores (*F*(1, 47) = 4.81, *p* = 0.033, *η*_*p*_^*2*^ = 0.095). Analyzing the full study data is crucial to determine the effectiveness of the NeuroNation MED application for MCI.

**Trial registration**

DRKS00025133, Date of registration: November 5, 2021.

**Supplementary Information:**

The online version contains supplementary material available at 10.1186/s40359-025-02458-w.

## Background

Mild cognitive impairment (MCI) is a condition in which individuals experience a decline in cognitive function in at least one cognitive domain compared to the respective age group [1]. MCI is commonly associated with limitations in complex activities of daily living [2] as well as lower quality of life, higher depressive and anxiety symptoms, and lower well-being [3]. The classification of MCI has undergone revisions over time. Initially, the focus was primarily on memory decline, often termed amnestic MCI (aMCI), which was frequently classified within the context of Alzheimer’s disease research [1]. Subsequently, the definition broadened to encompass impairments in other cognitive domains, leading to the classification of non-amnestic MCI (naMCI). This acknowledges the heterogeneity of MCI symptomatology, where deficits can affect either a single domain (single-domain MCI) or multiple domains (multi-domain MCI) [1, 4].

The diagnostic criteria employed in this study adhere to the ICD-10 classification (F06.7: Mild Cognitive Disorder) [5]. Unlike the DSM-5, the ICD-10 code F06.7 mandates an underlying physiological cause for an MCI diagnosis [4, 5]. Several factors can contribute to the development of MCI, including various neurodegenerative disorders, traumatic brain injuries, strokes, substance abuse [4, 6], and post-infectious conditions [7, 8]. The prevalence of MCI is generally higher in older adults [9], with aMCI being the most common subtype [10]. Older adults with aMCI face an increased risk of further cognitive decline and progression to Alzheimer’s dementia [11, 12]. However, given the diverse underlying etiologies, MCI can also occur in younger adults. Post-COVID-19-related cognitive decline cases exemplify this, affecting individuals across all age groups [7, 8, 13, 14]. In younger adults, as well as in older adults, MCI can lead to significant limitations in both social and work life [13, 15].

Considering the clinical relevance of MCI and the core symptom of impairment in at least one cognitive domain, the recommended treatment method is cognitive training (CT) with functional and strategy-oriented components [16]. CT is operationalized as repeated practice on standardized tasks and exercises that aim to stimulate specific cognitive domains and functions [17, 18]. Therefore, CT can be selectively applied to target specific impaired cognitive domains. Traditionally, CT is delivered as a face-to-face intervention involving a therapist and paper-and-pencil or computerized exercises. However, several limitations impede the widespread adoption of traditional CT for individuals with MCI. These limitations include long waiting times, mobility challenges, safety concerns during pandemics, physical limitations, and high treatment costs. Additionally, aging populations are expected to see a rise in MCI cases, while the number of available neuropsychological specialists is likely to remain stagnant [19]. As a result, mobile computerized CT (cCT) emerged as a potential treatment option due to its high accessibility via smartphones. cCT offers a convenient, self-administered, non-invasive, and personalized treatment approach. Individuals can engage in cCT exercises anywhere, including within the safe and private environment of their homes, overcoming many of the barriers associated with traditional CT [19]. Moreover, the gamified and tailored approach of cCT is accompanied by higher motivation and more consistent training adherence [20]. In recent years, the shift from traditional paper-and-pencil CT to cCT has led to a rapid growth of commercially available brain training software [21, 22]. However, with the growing number of commercially available cCT programs, many manufacturers have made exaggerated health claims with limited scientific and theoretical backing [23]. To establish cCT as a possible treatment option for MCI, well-controlled studies evaluating its effectiveness are essential.

A growing body of literature supports the potential of cCT. However, most empirical studies on cCT have limited generalizability, which restricts their applicability to individuals with MCI. These studies typically focus on healthy adults [21, 23–25], are not designed as randomized controlled trials (RCTs) [26], use blended cCT with supervision [27–29], or involve small sample sizes [19]. Additionally, studies that assess the effect of cCT on MCI often utilize varying diagnostic criteria [30]. All of these factors contribute to the current heterogeneity of the evidence on the effectiveness of mobile cCT for MCI [21]. Despite positive effects of cCT on global cognition [27, 31, 32], objective memory [27, 31–34], and subjective memory [35], findings on attention [28, 36], language, spatial perception, and executive functioning remain inconsistent [21, 27, 37]. While the primary focus of cCT research for individuals with MCI has been on its impact on cognitive abilities, meta-analyses suggest that cCT may also have positive effects on patient-related outcomes, such as depressive symptoms and quality of life [38, 39]. However, these findings have yet to be replicated [19]. Furthermore, MCI is associated with lower health literacy, which in turn limits individuals' ability to manage their health, including understanding medical information thoroughly, making informed decisions, and adhering to treatment plans [40]. In this context, interventions that promote self-management of therapy could positively impact health outcomes by empowering patients to take an active role in their treatment [41].

Recognizing these limitations regarding the effectiveness of cCT for individuals with MCI, methodological guidelines emphasize the need for large-scale RCTs in real-world settings to demonstrate effectiveness [26]. The NeuroNation MED Effectiveness study (NeNaE) was designed considering these methodological standards, examining the effectiveness of a specific mobile, self-administered gamified cCT program in a 12-week multicenter RCT [42]. However, conducting large-scale studies involves risks, both in terms of participant safety if treatments prove ineffective and resource investment in study execution [43, 44]. Interim analyses help address these concerns by enabling decisions about stopping a trial for futility, thus saving time, resources, and minimizing participant risks. Furthermore, a priori planned interim analyses should specify timing, stopping rules, and apply adequate alpha-adjustment methods to prevent Type 1 error inflation [44]. In the NeNaE protocol, a prespecified interim analysis with the first 50 participants was planned to assess early intervention effects on outcomes, evaluate potential risks or adverse effects, and decide whether to continue or terminate the study [42]. Hereby, this article presents the preliminary findings from this interim analysis, including the first 50 participants of the NeNaE, addressing the following research aims:

1. Exploring whether early effects are observable in both objective and subjective measures. Global cognition serves as the primary outcome measure, while attention, memory, language, visuospatial functions, and executive functions are secondary objective outcomes. Subjective patient-related measures, also considered secondary outcomes, comprised perceived cognitive functioning, psychosocial factors (e.g., depression, anxiety, psychological well-being, and self-efficacy), and health literacy.
2. Informing decisions about trial continuation, with negative or contrary findings leading to study termination [42].

## Methods

### Interim study design and setting

This interim analysis presents preliminary data obtained from the first 50 participants recruited for the full NeNaE [42]. The NeNaE aims to assess the effectiveness of a commercial gamified cCT, the NeuroNation MED medical device (MDD class I) for improving cognitive abilities in individuals with MCI [45]. The study was registered at the German Clinical Trials Register (DRKS00025133) as a multicenter RCT. All participants of the NeNaE and, thus, also of this interim analysis, gave their written consent and the Ethics Committee of the Charité – Universitätsmedizin Berlin approved the study (No. EA4/106/21).

### Study participants

#### Inclusion criteria

Individuals with a diagnosed F06.7 (“Mild Cognitive Disorder”) according to ICD-10 were included in the NeNaE. The MCI diagnosis was additionally validated by telephone screening using the Telephone Interview for Cognitive Status (TICS) [38]. Individuals with a TICS score between 21 and 32 were included. These score ranges include patients with both the ambiguous range (26 – 32) and the range of MCI (21 – 25) [46].

Furthermore, participants had to be at least 18 years old and able to independently understand the study information and provide informed consent. Since this study investigated the effectiveness of a digital intervention, all study participants had to have a mobile device with internet capability and be able to operate it independently. In addition, individuals were only included if they were able to sufficiently understand instructions in German.

#### Exclusion criteria

Individuals who scored greater than 32 or less than 21 on the TICS were excluded from study participation. This was due to the assumption that high scores above this range indicate normal cognitive functioning and that scores below this range indicate cognitive impairments too severe to follow study instructions and regimen. Furthermore, individuals with disabilities that could impair or limit app use were excluded, including those with paresis of the dominant arm or hand, visual field defects such as hemianopia or quadrantanopia, severe uncorrected or non-correctable visual impairments, as well as severe aphasia. Additionally, subjects were excluded from study participation if they were currently using other cCT programs.

##### Recruiting

**Fig. 1:**
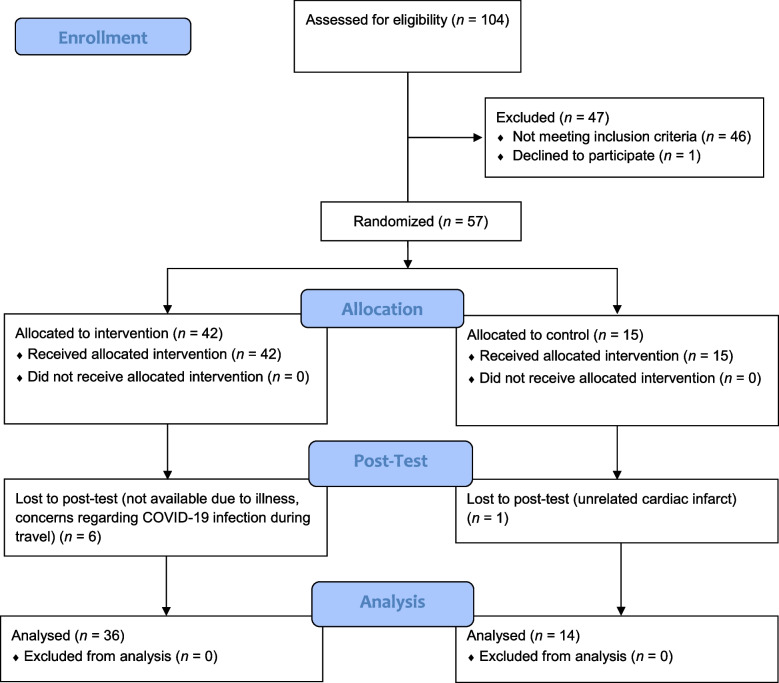
Initial CONSORT flow diagram of the interim analysis of the randomized controlled trial [47]

Participants were recruited from the Department of Geriatrics at Charité – Universitätsmedizin Berlin, the Department of Neurology at Charité – Universitätsmedizin Berlin, and the Department of Neurology at the University Hospital Jena. Local and regional advertising (including radio), along with emails, flyers, newsletters, and telephone calls, were used to recruit participants through neurological rehabilitation clinics and psychotherapists’ practices. As a result, interested individuals either contacted the study team directly or were approached by study personnel if they expressed a desire to participate or learn more about the study. Once a person indicated interest, a phone call was scheduled to provide detailed study information and present the inclusion and exclusion criteria. Eligible individuals were then given at least 24 h to consider participation before making a decision. All study participants were recruited, screened for inclusion criteria, and tested between 2021/10/01 and 2022/03/25. The detailed recruiting and screening procedure is outlined in the study protocol [42]. The data analysis took place in the Department of Geriatrics at Charité – Universitätsmedizin Berlin and in the Department of Neurology at the University Hospital Jena. The initial CONSORT flow diagram for the interim analysis is shown in Fig. 1.

### Sample included for the interim analysis

A total of 50 participants, consisting of an Intervention Group (IG; *n* = 36) and a Control Group (CG; *n* = 14) were included in this interim analysis (Table 1). Baseline demographics and clinical characteristics did not differ significantly between the two groups (Table 1). One serious adverse event, unrelated to the study, was reported in the CG during the study. Specifically, a participant was involved in a motor vehicle accident, resulting in minor injuries and short-term hospitalization. The participant confirmed that the accident was unrelated to their participation in the study.

**Table 1:** Interim analysis sample characteristics of the intervention group and the control group

Variables	IG (*n* = 36)	CG(*n* = 14)
Age in Years (*SD*)	58.1 (12.9)	59.6 (13.0)
Sex (%)		
Female	24 (66.7)	11 (78.6)
Male	12 (33.3)	3 (21.4)
Education (%)		
Apprenticeship	10 (27.8)	5 (35.7)
Technical College	4 (11.1)	1 (7.1)
University	15 (41.7)	3 (21.4)
Other	7 (19.4)	5 (35.7)
TICS (*SD*)	30.4 (2.2)	29.6 (2.6)

*TICS* Telephone interview for cognitive status

## Materials

In the following, the assessments for the primary and secondary outcomes are described. The primary outcome was the index score of the S-NAB. All other domains of the S-NAB, as well as the subjective assessments, were secondary outcomes.

### Telephone interview for cognitive status

The TICS, a cognitive screening test consisting of 11 items [48], was translated into German by two research associates of the Geriatrics Research group at Charité – Universitätsmedizin Berlin. To ensure accuracy and preserve the semantic concepts, the research associates created a single, consolidated version by carefully comparing both initial translations. However, two items needed slight modifications to better fit the German context. The item “Who is the President of the United States right now?” was changed to “Who is the Federal Chancellor of Germany right now?” and the item “Who is the Vice-President?” became “Who is the Federal President?” The translated TICS version was not validated statistically.

A total score of 41 points can be achieved. The cognitive performance can be classified based on the obtained score. The TICS was administered to a norm sample of 6726 individuals aged 18 and older, with approximately 94% of the total sample being over 60 years of age. The Split-Half reliability was *r* = 0.75. The validity of the TICS has been assessed and confirmed using several clinical samples [46].

### Neuropsychological assessment battery screening module

The S-NAB is a modular paper-and-pencil-based assessment that evaluates different cognitive domains using 14 neuropsychological subtests [49]. All study centers obtained the S-NAB licenses lawfully by purchasing the respective test sets, which included permission to use the S-NAB. These domains include attention, language, visuospatial functions, memory, and executive functions. An index score can be calculated to provide a measure of global cognition. Within the S-NAB, standardized and age-corrected scores are provided for all five modules, with a mean of 100 and a standard deviation of 15. Thus, scores between 85 and 114 represent average cognitive functioning, while scores ranging between 70 and 84 indicate a slight cognitive impairment in the respective domain [50]. The S-NAB norm sample consists of 880 adults aged 18 to 97 years. The reliability of the individual modules ranges from 0.70 to 0.93. Additionally, internal validity and criterion validity have been confirmed using clinical samples [51].

### Health-49 – Hamburg modules

The Health-49 questionnaire consists of 79 items in German, which are grouped into seven independent modules [52]. It assesses general aspects of mental health in therapeutic practice. In the present study, only Parts B and D – Psychological Well-being and Self-Efficacy – were included. A score ranging from 0 to 4 points can be achieved, with higher scores representing greater individual distress [52].

### Hospital anxiety and depression scale

The German version of the Hospital Anxiety and Depression Scale (HADS-D) is a self-assessment scale to determine the presence and severity of symptoms related to anxiety disorders and depression in patients [53, 54]. A score between 0 and 21 can be achieved in both subscales. An overall score can be calculated, ranging from 0 to 42. The higher the score, the more severe the psychological burden [55]. The HADS is considered reliable and consistent for both subscales, with Cronbach's alpha and split-half reliabilities both at 0.80.

### Health literacy questionnaire

The Health Literacy Questionnaire (HLQ) is a self-assessment tool for health literacy and patient sovereignty. For this study, the validated German translation was used (HLQ-D) [56]. This survey consists of 44 items divided into nine domains: 1. Feeling understood and supported by healthcare providers, 2. Having sufficient information to manage my health, 3. Actively managing my health, 4. Social support for health, 5. Appraisal of health information, 6. Ability to actively engage with healthcare providers, 7. Navigating the healthcare system, 8. Ability to find good health information, 9. Understanding health information well enough to know what to do. Questions can be answered using a four-point or five-point Likert scale. The HLQ-D survey is considered reliable, with Cronbach's alpha of at least 0.77.

### Cognitive failure questionnaire

The German version of the Cognitive Failure Questionnaire (CFQ-D) was used to assess the frequency of self-reported every day and transient errors related to memory, perception, and attention [57, 58]. The questionnaire consists of 32 items assessed with a five-point Likert scale each. A total score between 0 and 128 can be obtained. A higher score represents more reported everyday mistakes. The CFQ meets predictive and criterion validity, as well as reliability [59] and is suitable for individuals with cognitive disorders [60].

### Procedures

#### Study procedure and randomization

A face-to-face appointment was scheduled with screened potential participants. The baseline examination was identical for both the IG and the CG up to the randomization. If the individuals agreed to participate in the study, the consent form was signed. Subsequently, the S-NAB was conducted. After completing a questionnaire on sociodemographic characteristics, all other assessments were conducted.

To ensure comparability between the IG and the CG, stratified block randomization was performed in a 2:1 ratio, with blocks stratified by sex. Unbalanced randomization was used to ensure that more participants had access to the intervention, which was deemed appropriate for ethical reasons. This process was carried out at the end of the first study appointment by urn randomization with permuted block sizes. Subjects assigned to the IG received a user manual with access data to the NeuroNation MED application. We applied an intention-to-treat design in the study, meaning that all participants were included in the analysis regardless of their adherence to the recommended protocol. Participants were advised to use the app for three sessions per week, with one session taking 25 to 40 min. However, they could adjust the app usage time, either exceeding or reducing the recommended duration. The intervention period of twelve weeks was based on literature regarding the effectiveness of cCT programs [24].

The CG was a waiting group. Participants in this group did not receive any specific intervention but continued their usual ongoing treatment (if any) for 12 weeks. The post-test was conducted 12 weeks after the baseline assessment, with all participants undergoing a second round of testing using the S-NAB and all other assessments. In addition, the CG received access to the NeuroNation MED application after study completion.

#### Intervention: NeuroNation MED-application

**Fig. 2:**
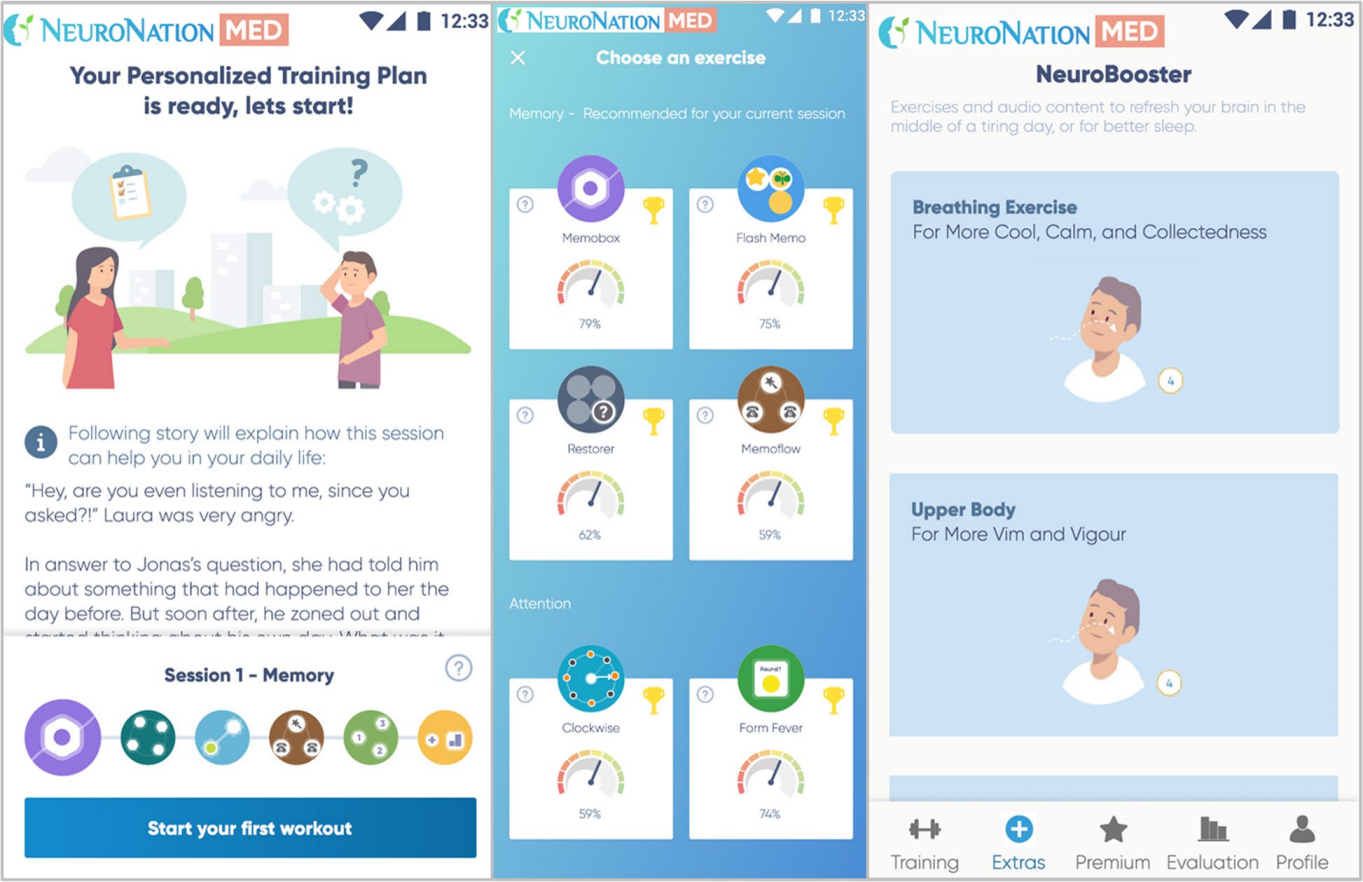
Exemplary screenshots of the NeuroNation MED application

The mobile application used in this study is NeuroNation MED, an adaptive, gamified multi-domain cCT specifically designed for individuals with MCI. The app is designed to train cognitive domains such as processing speed, executive functions, working memory, memory, attention, and verbal fluency through a variety of 23 exercises. The difficulty level adapts to the user's current performance and can also be manually adjusted. Each exercise is paired with practical, real-life storytelling examples, illustrating how potential transfer effects may occur within the training procedure. Immediate performance feedback, training reminders, and notifications aim to enhance user adherence. Short cognitive assessments during onboarding create a personalized cognitive profile used to generate an individual training plan. The training priorities for each cognitive domain can also be manually adjusted. NeuroNation MED also includes psychoeducation, with mental and physical practices designed to regulate emotions, promote relaxation, and improve concentration. Figure 2 shows exemplary screenshots of the NeuroNation MED application [45].

In the study, participants used the application on their own devices. The app was offered via Google's Play Store and Apple's App Store. Supported operating systems were iOS version 11.0 or Android version 5.0 or higher.

### Blinding

Due to the nature of the intervention, double blinding was not feasible. Thus, only single blinding of the study personnel was implemented. To minimize potential biases resulting from a lack of double blinding, objective assessments were used to measure cognitive abilities. Additionally, the baseline and post-tests were conducted by different study staff members to ensure the single-blinding process. Unblinding of the study staff took place only after the collection of all primary and secondary outcome variables at the end of the post-test.

### Sample size and power

The analysis presented here is an interim analysis, considering the first 50 subjects who completed both visits in the full NeNaE (see Table 1). A formal (sub-)sample size calculation for this interim analysis was not carried out as this analysis was purely exploratory. The detailed sample size calculation for the full NeNaE was calculated with G*Power 3 [61] and is illustrated in the published study protocol [42].

### Data analysis and statistical methods of the interim analysis

Data analyses for the primary and secondary outcomes were performed using IBM SPSS Statistics [62]. For the imputation of missing values, we used predictive mean matching using the MICE package in R [63, 64]. We imputed all missing values. Furthermore, we adjusted the alpha level to 0.00305 according to the O’Brien-Fleming method for this interim analysis. Effect sizes are reported as Partial Eta Squared, with effects classified as small (η_p_^2^ = 0.01), moderate (η_p_^2^ = 0.06), or large (η_p_^2^ = 0.14) and Cohen's *d* or Pearson correlation coefficient *r*, with effects classified as small (*d* = 0.2; *r* = 0.1), moderate (*d* = 0.5; *r* = 0.3), or large (*d* = 0.8; *r* = 0.5) [65, 66]. The Kolmogorov–Smirnov test and the Shapiro–Wilk test were applied to test for normal distributions of the outcome scores in baseline and post-test sessions. T-tests for paired samples were performed to compare baseline and post-test session scores of the S-NAB, Health-49, CFQ-D, and HADS-D within the IG and the CG. For the HLQ-D, the Wilcoxon signed-rank test was applied because no normal distribution was present. For group comparisons between the CG and IG, an Analysis of Covariance (ANCOVA) was performed. This involved sex, study center, as well as the experimental group (IG and CG) as fixed factors, baseline test session scores as a covariate and the post-test scores as the dependent variables [67, 68]. Appropriate prerequisite tests were applied before applying ANCOVA. In all assessments, the pre-test session results in the two groups did not differ from each other. ANCOVAs with corrections for baseline scores were calculated on the S-NAB, CFQ-D, HADS-D, and the Health-49.

## Results of the interim analysis

### Primary outcome: global cognition (S-NAB index score)

The interim analysis provided initial positive evidence for the effectiveness of the 12-week NeuroNation MED App-based cCT in the IG (Table 2). More specifically, a significant increase was found in the primary outcome, i.e., the S-NAB overall score reflecting the global cognition level with a mean difference of *MD* = 5.78 (*SD* = 10.94) (*M_*pre = 93.53, *SD_*pre = 16.45; *M_*post = 99.31, *SD_*post = 16.40; *t*(35) = 3.17, *p* = 0.0028, Cohen's *d* = 0.53) in the IG. In contrast, no evidence for a difference in the overall S-NAB score was found in the CG with *MD* = -0.79 (*SD* = 10.16) (*M*_pre = 91.64, *SD* pre = 14.88; *M_*post = 90.86; *SD_*post = 12.80; *t*(13) = 0.29, *p* = 0.777, Cohen's *d* = 0.08). The two-way ANCOVA determined a non-significant trend toward a difference between the groups on the S-NAB post-scores when controlling for S-NAB pre-scores. A non-significant trend towards higher improvement in the IG compared to the CG with a medium effect size in the index S-NAB score was found (*F*(1, 47) = 6.581, *p* = 0.014, η_p_^2^ = 0.125), which does not reach the adjusted alpha level of 0.00305.

**Table 2:** Initial Results of the global cognition and domains of the pre-post analysis of the S-NAB for the IG and CG interim analysis sample

	IG (*n* = 36)					CG (*n* = 14)				
	Pre	Post	*MD*	*p*-Value^††^	Cohen’s *d*	Pre	Post	*MD*	*p*-Value^††^	Cohen’s *d*
Global Cognition (*SD*)	93.5 (16.4)	99.3 (16.4)	5.8 (10.9)	.003^*^	0.53	92.1 (15.3)	90.1 (13.0)	-0.8 (10.2)	.777	0.08
Attention (*SD*)	88.4 (16.3)	94.8 (17.8)	6.4 (14.2)	.010	0.45	88.6 (18.3)	90.1 (17.4)	1.6 (11.8)	.625	0.13
Memory (*SD*)	99.7 (15.4)	99.3 (13.2)	-0.4 (16.0)	.893	0.02	100.1 (15.6)	96.4 (7.2)	-3.8 (17.0)	.419	0.22
Language (*SD*)	101.6 (17.8)	103.9 (12.7)	2.3 (18.5)	.459	0.13	91.2 (17.0)	95.6 (13.6)	4.4 (18.3)	.419	0.24
Visuospatial Functions (SD)	96.4 (19.0)	101.8 (19.1)	5.4 (20.3)	.121	0.27	100.0 (19.7)	97.9 (16.1)	-2.1 (22.2)	.724	0.10
Executive Functions (*SD*)	92.1 (16.9)	96.1 (16.1)	4.1 (13.8)	.087	0.29	92.5 (12.5)	89.9 (12.4)	-2.6 (10.4)	.372	0.25

Means including standard deviations for all cognitive domains assessed by the S-NAB

*Pre* baseline survey, *Post* Post-intervention measurement, *MD* Mean difference between the between pre- and post-score

^*^p < .00305—alpha level for this interim analysis

^††^*t*-test for paired samples of the mean value of the IG and the mean value of the CG in a pre-post comparison

### Secondary outcomes

The secondary outcomes included the S-NAB domain sub-scores (see Table 2) and patient-related outcomes, i.e., subjective cognitive functioning, psychosocial constructs, and health literacy (see Supplementary File 1).

#### Cognitive domains: attention, language, memory, spatial ability, and executive functions (S-NAB domains)

For the domain-specific subscores, none of the pre-post data comparisons revealed significant effects in the IG or the CG, likely due to the limited sample size of the interim analysis. Group comparisons in the subdomains on the S-NAB post-subscores, controlling for S-NAB pre-subscores, were not statistically significant: attention (*F*(1, 47) = 1.505, *p* = 0.226, η_p_^2^ = 0.032), language (*F*(1, 47) = 2.131, *p* = 0.151, η_p_^2^ = 0.044), memory (*F*(1, 47) = 1.137, *p* = 0.292, η_p_^2^ = 0.024), spatial ability (*F*(1, 47) = 1.638, *p* = 0.207, η_p_^2^ = 0.034), executive functions (*F*(1, 47) = 3.837, *p* = 0.056, η_p_^2^ = 0.077).

#### Subjective cognitive functioning (CFQ-D)

The IG showed a significant mean decrease in everyday mistakes measured with the CFQ-D (*MD* = -10.56, *SD* = 18.82, *t*(35) = 3.21, *p* = 0.0029, Cohen's *d* = 0.53). In the CG, no evidence for such a decrease was observed (*MD* = -2.00, *SD* = 11.67, *t*(13) = 0.64, *p* = 0.532). A two-way ANCOVA did not reveal a significant group difference in the CFQ-D post-test session scores between the groups when controlling for the pre-test session (*F*(1, 47) = 4.478, *p* = 0.040, η_p_^2^ = 0.089).

#### Depressive and anxiety symptoms (HADS-D)

No significant differences were found between the pre- and the post-test session values in the HADS-D total score in the IG (*MD* = -1.39, *SD* = 5.07, *t*(35) = 1.65, *p* = 0.109) or the CG (*MD* = 2.00, *SD* = 4.80, *t*(13) = 1.56, *p* = 0.143). In a two-way ANCOVA, no significant group difference was found when controlling for the pre-test session scores after alpha adjustment (*F*(1, 47) = 4.815, *p* = 0.033, η_p_^2^ = 0.095). We also did not find significant effects for the subscale i.e., depression symptoms (*F*(1, 47) = 1.245, *p* = 0.029, η_p_^2^ = 0.099) or anxiety symptoms (*F*(1, 47) = 2.430, *p* = 0.126, η_p_^2^ = 0.050).

#### Self-efficacy and well-being (Health-49)

Paired-samples *t*-tests were used to compare the two subscale scores of the Health-49, Psychological Well-being and Self-Efficacy, between the post- and the pre-assessment. Within the IG, no evidence for a change in the Psychological Well-being scale score was found (*MD* = -0.48, *SD* = 1.17, *t*(35) = 2.46, *p* = 0.019), while the changes for the self-efficacy scale were on the edge of significance: (*MD* = -0.56, *SD* = 1.07, *t*(35) = 3.14, *p* = 0.003, Cohen's *d* = 0.52). Within the CG, no evidence for differences in either of the two scale scores was found (Psychological Well-being: *MD* = -0.06, *SD* = 1.26, *t*(13) = 0.19, *p* = 0.852; Self-Efficacy: *MD* = -0.19, *SD* = 0.73, *t*(13) = 0.95, *p* = 0.361).

A two-way ANCOVA revealed no evidence for group differences in the post-test session when controlling for baseline values (Psychological Well-being: *F*(1, 47) = 0.862,* p* = 0.358, η_p_^2^ = 0.018; Self-Efficacy: *F*(1, 47) = 3.189, *p* = 0.081, η_p_^2^ = 0.065).

#### Health literacy (HLQ-D)

Wilcoxon signed-rank tests were conducted to compare the HLQ-D subscales in the post- to the pre-assessment. Within both the IG and CG, no differences were found (see Supplementary File 1). A two-way ANCOVA revealed no evidence for group differences in the post-test session when controlling for baseline values for any HLQ-D subscale [Subscale 1: (*F*(1, 47) = 4.636, *p* = 0.037, ηp2 = 0.092); 2: (*F*(1, 47) = 3.068, *p* = 0.087, ηp2 = 0.063); 3: (*F*(1, 47) = 0.024, *p* = 0.877, ηp2 = 0.001); 4: (*F*(1, 47) = 0.646, *p* = 0.426, ηp2 = 0.014); 5: *(F*(1, 47) = 3.864, *p* = 0.055, ηp2 = 0.077); 6: (*F*(1, 47) = 5.773, *p* = 0.020, ηp2 = 0.112); 7: (*F*(1, 47) = 2.142, *p* = 0.150, ηp2 = 0.044); 8: (*F*(1, 47) = 2.724, *p* = 0.106, ηp2 = 0.057); 9: (*F*(1, 47) = 9.583, *p* = 0.003, ηp2 = 0.172)].

## Discussion

This research article presents preliminary interim outcomes from the NeNaE, evaluating a mobile, self-administered cCT program in adults with MCI over a 12-week RCT. These initial results indicate that self-administered cCT may improve cognitive status and subjective cognitive functioning in the IG. Specifically, pre-post comparisons demonstrated improvements in global cognition and subjective cognitive functioning within the IG. Notably, statistical tests did not yet show differences between the IG and the CG. Nevertheless, effect sizes indicated a medium treatment effect on global cognition and small effects on other cognitive domains. These preliminary findings may suggest potential benefits of NeuroNation MED, but require cautious interpretation due to the limited sample size and unbalanced allocation ratio. The prespecified 2:1 allocation ratio (IG to CG) [42] could not be realized for this interim analysis due to the randomization process. Additionally, the pre-fixed analysis only included the first 50 participants recruited to the full study. This may bias results, underscoring the need for the complete study to fully assess NeuroNation MED's effectiveness.

Still, these preliminary findings align with previous reports of small to moderate effects of cCT on global cognition in MCI [19, 27, 31, 32, 35]. While empirical findings have shown positive treatment effects of cCT on general cognitive functioning, its effectiveness on specific cognitive domains remains less clear [19, 21].In this exploratory analysis, we obtained preliminary indications of effects on global cognition. However, results from the complete sample are necessary to determine the effectiveness of NeuroNation MED in improving both global cognition and domain-specific cognitive performance. Many cCT interventions have focused on the amelioration of memory and attention deficits [21, 23, 69]. Interestingly, an improvement in subjective memory was observed in the IG. This is consistent with the research by Bahar-Fuchs et al. [35], where a home-based, adaptive cCT program led to improvements in subjective memory in individuals with MCI. Despite the increasing availability of mobile cCT programs and various health claims suggesting that they can lead to far transfer and enhance activities of daily living, improve mental health and positively impact overall quality of life [23], there is a lack of empirical evidence to support these assertions [19]. While our interim analysis did not find improvements in depressive and anxiety symptomatology, well-being, self-efficacy, or health literacy, this might be due to the lack of statistical power. It will only be possible to determine whether the intervention actually leads to treatment effects on these patient-related outcomes after the complete study data are analyzed.

In conclusion, cCT is a low-resource intervention characterized by accessibility, versatility, and cost-effectiveness, which may have a positive effect on cognition. Moreover, mobile cCT presents the advantage of increasing its reach and access to healthcare and potentially reducing overall treatment costs. The findings of this interim analysis provide first evidence in favor of the assumption that global cognition may be improved by the cCT. To confirm the effectiveness of the mobile cCT – NeuroNation MED – in individuals with MCI, the large sample of the complete study is necessary, given the generally small to medium effect sizes of cCT [24, 34, 38].

### Limitations

This interim analysis presents several limitations. Primarily, the small sample size limits the statistical power to detect significant effects, increasing the risk of Type II errors. Consequently, non-significant findings may not accurately reflect the intervention's true effectiveness. Additionally, the unbalanced allocation ratio in this interim sample may introduce allocation bias, potentially skewing the results. The inability to differentiate between MCI subtypes (aMCI vs. naMCI) is a consequence of the NeNaE study design [42] and restricts the generalizability of the findings across different MCI populations. Furthermore, the absence of an active control group limits the ability to attribute observed effects solely to the NeuroNation MED intervention, as placebo effects or external factors could influence outcomes. Potential confounding variables, such as variations in training adherence, medication use, physical activity, and overall health status, were not controlled in this interim analysis. These factors could independently affect cognitive outcomes and were not assessed due to the study's preliminary nature. Lastly, the TICS was administered using a self-translated German version, which may compromise measurement invariance and construct validity, potentially introducing semantic inconsistencies and affecting the reliability of cognitive assessments.

## Conclusion

These initial results of the interim analysis provide first insights into the field of mobile cCT. The results suggest that mobile cCT may have the potential to improve global cognition and subjective memory. However, only the full study data, including the complete sample, will allow us to analyse whether these exploratory results are replicable and whether treatment effects on other cognitive domains will also be found. CCT offers a low-cost and non-invasive treatment option with the potential to enhance both objective and subjective cognitive function in MCI. While we did not find evidence for an obvious impact of cCT on psychosocial functions in this preliminary analysis including a small sample, the full sample analyses could still reveal subtle effects on depressive and anxiety symptomatology, well-being, and self-efficacy. The completion of the full data analysis will provide conclusive results on the effectiveness of NeuroNation MED in improving cognitive functioning and patient-related outcomes in adults with MCI.

## Supplementary Information

Supplementary Material 1. Initial results of the pre-post interim analysis of the secondary outcomes for the IG and CG.

## Data Availability

The datasets analysed during this interim analysis are not publicly available so that the anonymity of participants involved is preserved. The dataset may be available from the corresponding author on reasonable request.

## Abbreviations

ANCOVA: Analysis of Covariance
aMCI: Amnestic MCI
CT: Cognitive Training
cCT: Computerized Cognitive Training
CG: Control Group
CFQ-D: German Version of the Cognitive Failure Questionnaire
HLQ-D: German Version of the Health Literacy Questionnaire
HLQ: Health Literacy Questionnaire
HADS-D: Hospital Anxiety and Depression Scale
IG: Intervention Group
MCI: Mild Cognitive Impairment
NeNaE: NeuroNation MED Effectiveness Study
S-NAB: Neuropsychological Assessment Battery Screening Module
naMCI: Non-Amnestic MCI
RCTs: Randomized Controlled Trials
TICS: Telephone Interview for Cognitive Status
