# Supplementary material for: Preliminary effects of mobile computerized cognitive training in adults with mild cognitive impairment: interim analysis of a randomized controlled trial
Source: BMC Psychol. 2025 Mar 5;13:202. doi: 10.1186/s40359-025-02458-w (PMC11881253; doi:10.1186/s40359-025-02458-w)
Supplement: Supplementary file 1 [file 40359_2025_2458_MOESM1_ESM.odt]

Supplementary File 1

Initial results of the pre-post interim analysis of the secondary outcomes for the IG and CG

 

|  | IG (n =36) |  |  | CG (n =14) |
| --- | --- | --- | --- | --- |
|  | Pre | Post | MD | p-Value†† | Effect size |  |  | Pre | Post | MD | p-Value†† | Effect size |
| CFQ-D (SD) | 52.33 (20.31) | 42.28 (19.51) | - 10.56 (18.82) | .003 | 0.53§ |  |  | 57.50 (16.61) | 55.50 (18.29) | - 2.00 (11.67) | .532 | 0.17§ |
| HADS-D             Total Score (SD) | 13.28 (5.63) | 11.89 (6.04) | - 1.39 (5.07) | .109 | 0.27§ |  |  | 13.14 (9.12) | 15.14 (9.04) | 2.00 (4.80) | .143 | 0.42§ |
| HADS-D        Anxiety (SD) | 6.64 (3.21) | 6.22 (3.20) | - 0.48 (1.17) | .474 | 0.12§ |  |  | 7.29 (5.33) | 8.07 (4.62) | 0.79 (3.09) | .359 | 0.25§ |
| HADS-D Depression (SD) | 6.64 (3.22) | 5.67 (3.91) | -0.97 (2.93) | .055 | 0.33§ |  |  | 5.86 (4.15) | 7.07 (4.73) | 1.21 (2.78) | .126 | 0.44§ |
| Health-49 Well-being (SD) | 2.06 (1.22) | 1.58 (0.73) | - 0.48 (1.17) | .019 | 0.41§ |  |  | 1.86 (1.04) | 1.79 (0.89) | - 0.06 (1.26) | .852 | 0.05§ |
| Health-49 Self-Efficacy (SD) | 1.97 (1.03) | 1.41 (0.82) | - 0.56 (1.07) | .003 | 0.52§ |  |  | 2.01 (1.14) | 1.83 ( 0.75) | - 0.19 (0.73) | .361 | 0.25§ |
| HLQ-D               Subscale 1 (SD) | 3.06 (0.47) | 3.13 (0.42) | 0.06 (0.44) | .348 | 0.11† |  |  | 3.16 (0.57) | 2.88 (0.71) | -0.29 (0.62) | .136 | 0.28† |
| HLQ-D               Subscale 2 (SD) | 2.71 (0.51) | 2.77 (0.53) | 0.06 (0.45) | .331 | 0.12† |  |  | 2.57 (0.58) | 2.46 (0.38) | -0.11 (0.59) | .538 | 0.12† |
| HLQ-D               Subscale 3 (SD) | 2.92 (0.38) | 2.96 (0.40) | 0.04 (0.43) | .459 | 0.09† |  |  | 2.96 (0.63) | 3.01 (0.43) | 0.06 (0.59) | .594 | 0.10† |
| HLQ-D               Subscale 4 (SD) | 2.97 (0.52) | 2.98 (0.53) | 0.01 (0.49) | .743 | 0.04† |  |  | 2.81 (0.56) | 2.74 (0.63) | -0.07 (0.38) | .435 | 0.15† |
| HLQ-D               Subscale 5 (SD) | 2.64 (0.56) | 2.82 (0.50) | 0.17 (0.50) | .047 | 0.34* |  |  | 2.70 (0.40) | 2.57 (0.69) | -0.13 (0.46) | .259 | 0.21† |
| HLQ-D               Subscale 6 (SD) | 3.52 (0.63) | 3.58 (0.62) | 0.06 (0.57) | .372 | 0.11† |  |  | 3.31 (0.89) | 3.01 (1.02) | -0.30 (0.59) | .092 | 0.59* |
| HLQ-D               Subscale 7 (SD) | 3.51 (0.54) | 3.53 (0.64) | 0.23 (0.08) | .811 | 0.03† |  |  | 3.19 (0.83) | 3.08 (0.66) | -0.11 (0.61) | .581 | 0.10† |
| HLQ-D               Subscale 8 (SD) | 3.44 (0.69) | 3.55 (0.66) | 0.11 (2.93) | .183 | 0.16† |  |  | 3.34 (0.58) | 3.23 (0.81) | -0.11 (0.58) | .306 | 0.58§ |
| HLQ-D               Subscale 9 (SD) | 3.58 (0.73) | 3.77 (0.63) | 0.19 (0.40) | .011 | 0.30† |  |  | 3.46 (0.56) | 3.31 (0.62) | -0.14 (0.56) | .324 | 0.56§ |

Note. Means including standard deviations for the assessments CFQ-D, Health-49 and HLQ-D. Pre = baseline survey, Post = post-intervention measurement, MD = difference between pre- and post-score, effect sizes: † r, § Cohen’s d. ††t-test for paired samples of the mean value of the IG and the mean value of the CG in a pre-post comparison.
